# A nomogram model based on HALP score and sST2 for predicting 1-year MACE risk after PCI in acute myocardial infarction patients

**DOI:** 10.3389/fcvm.2025.1641855

**Published:** 2025-09-03

**Authors:** Chen-Yan Li, Hai-Bo Wu, Ya-Wei Duan, Peng Gao, Hong-Xiao Li, Xue-Chao Wang, Yun-Can Wang, Yan-Qing Wang, Shi-Ru Bai, Yuan Jia, Rong-Pin Du

**Affiliations:** ^1^ Hebei North University, Zhangjiakou, Hebei, China; ^2^ Hebei General Hospital, Shijiazhuang, China; ^3^ Department of Ultrasound, The First Hospital of Hebei Medical University, Shijiazhuang, Hebei, China

**Keywords:** acute myocardial infarction, HALP score, sST2, nomogram, MACE events, post-PCI

## Abstract

**Objective:**

To develop a nomogram model integrating the HALP score (a composite score of hemoglobin, albumin, lymphocytes, and platelets) and sST2 for predicting the risk of major adverse cardiovascular events (MACE) within 1 year after percutaneous coronary intervention (PCI) in patients with acute myocardial infarction (AMI).

**Methods:**

This retrospective analysis included 236 AMI patients undergoing emergency PCI (2019–2024), categorized into MACE (*n* = 102) and non-MACE (*n* = 134) groups. Independent predictors were identified through multivariate logistic regression analysis, and a nomogram model was constructed. Model performance was validated using receiver operating characteristic (ROC) curves and the Bootstrap method (*N* = 1,000).

**Results:**

Multivariate analysis revealed that Killip class IV (OR = 3.758, *P* *=* 0.009), high sST2 levels (OR = 1.008, *P* *=* 0.009), high LDL-C (OR = 1.533, *P* *=* 0.041), high LVEDD (OR = 1.106, *P* *=* 0.009), and low HALP score (OR = 0.958, *P* *=* 0.023) were independent predictors of MACE. The combined model exhibited significantly better predictive performance than single indicators (AUC = 0.833, 95% CI: 0.781–0.886), with a sensitivity of 87.3% and specificity of 68.7%. The nomogram demonstrated good calibration after Bootstrap validation (Hosmer-Lemeshow test *P* *=* 0.157).

**Conclusion:**

The nomogram model developed in this study, which integrates the HALP score (reflecting inflammatory-nutritional status) and sST2 (a marker of myocardial fibrosis) along with clinical indicators, can effectively predict the risk of MACE after PCI and provides a visual tool for individualized risk stratification.

## 1 Introduction

Cardiovascular diseases (CVD) remain the leading cause of morbidity and mortality worldwide, affecting not only individual health but also imposing a substantial economic burden on global healthcare systems. The 2023 World Heart Report indicates that in 2021, 20.5 million people died from cardiovascular diseases. Among these, myocardial infarction (MI) is still the primary cause of death (1, 2). The optimal strategy for managing myocardial infarction is the rapid implementation of myocardial reperfusion, and percutaneous coronary intervention (PCI) is currently one of the most important therapeutic approaches. It can restore blood flow to the infarcted myocardium, thereby reducing myocardial damage and improving patient outcomes (3–6). However, approximately 10% to 15% of patients still experience major adverse cardiovascular events (MACE) after PCI, significantly increasing the risk of death (7). Therefore, identifying reliable predictors of MACE risk in patients with acute myocardial infarction (AMI) after PCI is of great clinical significance.

Current research suggests that inflammatory and nutritional indicators are associated with the prognosis of myocardial infarction. Additionally, other studies have identified independent predictors of myocardial infarction prognosis, including gender, age, Killip classification, infarct location, left ventricular ejection fraction (LVEF), and peak CK-MB levels (8–11). Our study aims to build on previous research by exploring a novel, simple, and convenient model for predicting the risk of MACE events within one year after PCI in patients with acute myocardial infarction.

The HALP score, calculated based on hemoglobin, albumin, lymphocyte, and platelet levels, is a simple and convenient indicator that integrates inflammatory and nutritional status into a comprehensive score. Previous studies have demonstrated that the HALP score can predict the prognosis of patients with various types of cancer, particularly gastric, pancreatic, and prostate cancers, as well as stroke patients (12–15). The formula for calculating the HALP score is: hemoglobin (g/L) × albumin (g/L) × lymphocyte count (10^9^/L)/platelet count (10^9^/L). Recent research has shown that it is associated with adverse cardiovascular outcomes (16).

ST2, as a member of the interleukin-1 receptor family, plays a key role in the mechanical stress response and fibrosis process of the myocardium (17). Although the primary cellular source of ST2 has not been definitively identified, evidence suggests that vascular endothelial cells may be an important source of ST2. The transmembrane receptor ST2l and the soluble receptor sST2 are two key subtypes of the ST2 protein. Among them, soluble ST2 (sST2) is released into the bloodstream, acting as a decoy receptor for IL-33, thereby blocking the signaling between IL-33 and ST2l and its beneficial effects. The interaction between sST2 and IL-33 is closely related to the development and progression of various inflammatory diseases, cardiac pathological conditions, and cancers (18, 19). Currently, risk models guided by sST2 are mostly focused on heart failure research, where adverse outcomes in heart failure patients may be associated with elevated sST2 levels, making it a potential novel risk factor for predicting poor prognosis (20). However, there are few studies on the predictive efficacy of sST2 for MACE events after PCI in acute myocardial infarction, and the synergistic effect with emerging inflammatory-nutritional indicators, such as the HALP score, needs to be verified.

This study aimed to develop and validate a novel nomogram model for predicting the risk of MACE within 1 year after percutaneous coronary intervention (PCI) in patients with AMI. Through a single-center retrospective analysis of 236 AMI patients (102 in the MACE group vs. 134 in the non-MACE group), the study focused on the combined predictive value of two emerging biomarkers—metabolic-inflammation imbalance (HALP score) and myocardial fibrosis (sST2)—and integrated clinical indicators [such as Killip classification and left ventricular end-diastolic diameter (LVEDD)] to construct a personalized risk assessment tool more suitable for the East Asian population. This tool aims to guide risk stratification and precision intervention for patients after PCI. The innovations of this study include: (1) the first validation of the HALP score's value in predicting MACE after PCI, addressing the neglect of metabolic-inflammation interactions in traditional scoring systems; (2) combining the nutritional-inflammation marker HALP score with myocardial fibrosis biomarker sST2, filling a gap in existing risk prediction models that overlook the interaction between metabolic-inflammation status and myocardial remodeling processes; and (3) developing a clinically translatable nomogram model. After Bootstrap validation, the model outperformed single indicators, providing clinicians with an intuitive MACE risk stratification scheme.

## 2 Materials and methods

### 2.1 Study population

This study retrospectively included 236 patients with AMI who were initially diagnosed and underwent emergency PCI at Hebei Provincial People's Hospital from January 2019 to January 2024. Of these patients, 180 were male. The inclusion criteria were as follows: (1) Patients with AMI meeting the criteria of the Fourth Universal Definition of Myocardial Infarction; (2) Patients undergoing emergency PCI; (3) Patients with complete data and follow-up. The exclusion criteria were as follows: (1) Previous history of AMI or coronary artery bypass grafting (CABG); (2) Presence of malignancy; (3) Severe hepatic dysfunction (defined as ALT >3×ULN) or renal impairment (eGFR <30 ml/min/1.73m² using CKD-EPI equation; (4) Incomplete medical records or inability to cooperate with treatment; (5) Presence of autoimmune diseases, etc.Patients were divided into the MACE group (*n* = 102) and the non-MACE group (*n* = 134) based on whether MACE occurred within 1 year after the procedure. In this study, MACE was defined as a composite endpoint of cardiovascular death, stroke, heart failure (new-onset heart failure and worsening of existing heart failure), and ischemia-driven revascularization within 1 year after PCI for acute myocardial infarction. The study strictly adhered to the ethical principles of the Declaration of Helsinki and was approved by the Ethics Committee of Hebei General Hospital (ethical approval No.: 2025-KY42).

### 2.2 General clinical data

Clinical data of the included patients were collected through the hospital's medical record system. The collected data included the following:

• Demographic and lifestyle factors: Gender, age, smoking history.
• Cardiovascular disease-related factors: Infarct location, number of diseased vessels, number of stents implanted, hypertension, diabetes, Killip classification.
• Laboratory parameters:
• Cardiac biomarkers: NT-proBNP, CK-MB, cardiac troponin T (cTnT).
• Metabolic parameters: Fasting blood glucose, lipoprotein a, triglycerides, total cholesterol, low-density lipoprotein cholesterol (LDL-C), hemoglobin, albumin.

Hematologic parameters: Neutrophils, lymphocytes, monocytes, platelets, serum creatinine.

(1) Inflammatory and nutritional indices:
  (1) Neutrophil-to-lymphocyte ratio (*N*LR).
  (2) Systemic immune-inflammation index (SIRI): Neutrophil count × Monocyte count/Lymphocyte count.
  (3) Prognostic nutritional index (PNI): Serum albumin (g/L) + 5 × Peripheral blood lymphocyte count (×10^9^/L).
  (4) PLR: Platelet count/Lymphocyte count.

### 2.3 Echocardiographic parameters

All patients in this study underwent echocardiography using a Philips Color Doppler Ultrasound Diagnostic Instrument (Philips Healthcare, Netherlands) to measure the LVEDD and LVEF. Measurements were averaged over three cardiac cycles.

### 2.4 Calculation of HALP score and collection of sST2

#### 2.4.1 Calculation of HALP score

The HALP score was calculated using the following formula:

HALP *=* hemoglobin (g/L) × albumin (g/L) × lymphocyte count (10^9^/L)/platelet count (10^9^/L)

All parameters were measured within 24 hours of admission and before PCI.

#### 2.4.2 Measurement of sST2

The sST2 levels were measured using venous blood samples collected within 24 hours of admission and before PCI. These samples were sent to the laboratory for testing. The measurements were conducted using a chemiluminescence immunoassay with an automated chemiluminescence analyzer (Chongqing Keximai Co., Ltd.: Model: SMART 300) by the Hebei Provincial People's Hospital laboratory. In this experiment, the threshold for the sST2 standard kit was set at 35 ng/ml (results under 35 ng/ml are considered negative). Data were sourced from the medical records system of Hebei Provincial People's Hospital and were retrospectively tracked. The final data included were from patients with acute myocardial infarction who had complete records of sST2 measurements.

### 2.5 Statistical methods

The statistical analysis of the study data was performed using SPSS version 26.0. For continuous variables with a normal distribution, data were described using the mean ± standard deviation ($\bar{x}\pm s$) and compared between groups using the independent samples *t*-test. For continuous variables that did not conform to a normal distribution, data were described using the median and interquartile range (M Q1, Q3) and compared between groups using the Mann–Whitney *U*-test. Categorical variables were described using counts and percentages (%), and comparisons between groups were made using the chi-square test or Fisher's exact test when appropriate.

Variables that showed significant differences between groups in univariate analysis were included in multivariate logistic regression analysis to identify independent predictors of MACE. Multicollinearity was assessed using variance inflation factors (VIF < 5 considered acceptable). The missing values in this data are less than 2%. The method of directly deleting the cases with missing values is adopted. The diagnostic performance of these predictors was evaluated using receiver operating characteristic (ROC) curves to assess their independent and combined predictive abilities. The nomogram model was constructed using R software (version 4.1.0) with the rms package. The discrimination and calibration of the nomogram model were assessed using the area under the ROC curve (AUC) and calibration curves, respectively. A *p*-value of less than 0.05 was considered statistically significant.

## 3 Results

### 3.1 Baseline comparison between MACE and non-MACE groups

A total of 236 patients with AMI who underwent PCI were included in this study. Of these, 102 patients (43.22%) were in the MACE group and 134 patients (56.78%) were in the non-MACE group. Baseline comparisons between the two groups revealed significant differences in the following variables:

• Killip classification (*P* < 0.001)
• sST2 levels (*P* < 0.001)
• Neutrophil count (*P* *=* 0.031)
• Serum creatinine (*P* *=* 0.005)
• LDL-C (*P* *=* 0.038)
• LVEDD (*P* *=* 0.005)
• HALP score (*P* < 0.001)
• SIRI (*P* < 0.001)
• NLR (*P* < 0.001)
• PNI (*P* < 0.001)
• Platelet-to-lymphocyte ratio (PLR) (*P* < 0.001)

All these differences were statistically significant (*P* < 0.05).

Specifically, for Killip classification, the proportion of patients with Killip class IV was significantly higher in the MACE group (20.59%) than in the non-MACE group (5.22%). For sST2, the median level in the MACE group (87.51) was significantly higher than that in the non-MACE group (37.53). For neutrophils, the median count in the MACE group (8.27) was significantly higher than that in the non-MACE group (7.30). For serum creatinine, the median level in the MACE group (80.80) was significantly higher than that in the non-MACE group (71.60). For LDL-C, the median level in the MACE group (3.47) was significantly higher than that in the non-MACE group (3.29). For LVEDD, the median value in the MACE group (49.00) was significantly higher than that in the non-MACE group (47.00). For HALP score, the median value in the MACE group (26.62) was significantly lower than that in the non-MACE group (39.76). For SIRI, the median value in the MACE group (3.43) was significantly higher than that in the non-MACE group (2.02). For NLR, the median value in the MACE group (6.20) was significantly higher than that in the non-MACE group (4.49). For PNI, the median value in the MACE group (43.50) was significantly lower than that in the non-MACE group (47.08). For PLR, the median value in the MACE group (183.84) was significantly higher than that in the non-MACE group (131.77). The detailed results are presented in Table 1.

**Table 1:** Comparison of relevant indicators between Non-MACE and MACE groups.

Characteristics	MACE not occurred (*n* = 134)	MACE occurred (*n* = 102)	Z/x²	*P*
Infarct location			2.981	0.395
Lateral wall	2 (1.49%)	2 (1.96%)		
Inferior wall	46 (34.33%)	27 (26.47%)		
Inferoposterior wall	23 (17.16%)	14 (13.73%)		
Anterior wall	63 (47.01%)	59 (57.84%)		
Gender			0.746	0.388
Female	29 (21.64%)	27 (26.47%)		
Male	105 (78.36%)	75 (73.53%)		
Smoking			0.323	0.570
No	62 (46.27%)	51 (50.00%)		
Yes	72 (53.73%)	51 (50.00%)		
Hypertension			3.578	0.059
No	61 (45.52%)	34 (33.33%)		
Yes	73 (54.48%)	68 (66.67%)		
Diabetes			3.593	0.058
No	103 (76.87%)	67 (65.69%)		
Yes	31 (23.13%)	35 (34.31%)		
Killip classification			21.790	<0.001
Class I	80 (59.70%)	36 (35.29%)		
Class II	44 (32.84%)	38 (37.25%)		
Class III	3 (2.24%)	7 (6.86%)		
Class IV	7 (5.22%)	21 (20.59%)		
Number of diseased vessels			2.957	0.228
Single vessel	44 (32.84%)	24 (23.53%)		
Double vessel	43 (32.09%)	33 (32.35%)		
Triple vessel	47 (35.07%)	45 (44.12%)		
Number of stents implanted	1.00 (1.00, 2.00)	1.00 (1.00, 2.00)	−1.549	0.121
Age (years)	58.00 (47.00, 68.00)	60.00 (52.00, 70.00)	−1.631	0.103
sST2 (pg/ml)	37.53 (25.33, 58.09)	87.51 (42.44, 147.37)	−6.093	<0.001
CK-MB (IU/L)	42.40 (26.20, 72.00)	41.85 (17.20, 87.50)	−0.038	0.969
Troponin T (ng/L)	2,000.00 (724.00, 2,000.00)	2,000.00 (823.00, 2,000.00)	−0.297	0.766
Neutrophils (10^9^/L)	7.30 (5.86, 10.03)	8.27 (6.44, 11.13)	−2.157	0.031
Serum creatinine (μmol/L)	71.60 (62.30, 84.90)	80.80 (66.70, 96.50)	−2.791	0.005
Total cholesterol (mmol/L)	4.83 (4.20, 5.45)	4.94 (4.18, 5.55)	−0.486	0.627
Triglycerides (mmol/L)	1.40 (0.94, 2.23)	1.44 (0.99, 1.87)	−0.579	0.562
Lipoprotein a (mg/L)	180.00 (116.10, 308.40)	195.95 (90.60, 333.90)	−0.205	0.838
LDL-C (mmol/L)	3.29 (2.73, 3.71)	3.47 (2.97, 3.90)	−2.072	0.038
LVEF (%)	54.00 (48.00, 59.00)	56.00 (47.00, 60.00)	−0.618	0.536
LVEDD (mm)	47.00 (45.00, 50.00)	49.00 (46.00, 53.00)	−2.830	0.005
HALP score	39.76 (28.34, 53.46)	26.62 (17.48, 34.43)	−6.074	<0.001
SIRI	2.02 (1.22, 3.17)	3.43 (1.92,5.14)	−4.500	<0.001
NLR	4.49 (2.92, 7.02)	6.20 (4.18,10.71)	−4.090	<0.001
PNI	47.08 (43.55, 50.25)	43.50 (39.70,46.20)	−5.751	<0.001
PLR	131.77 (99.09, 185.33)	183.84 (136.81, 244.51)	−4.909	<0.001

### 3.2 Factors influencing MACE occurrence—multivariate logistic regression analysis

We constructed a multivariate logistic regression model using the occurrence of MACE (no = 0, yes = 1) as the dependent variable and the significant factors identified in the univariate analysis—Killip classification, sST2, neutrophils, serum creatinine, LDL-C, LVEDD, HALP score, SIRI, NLR, PNI, and PLR—as independent variables.

The results are shown in Table 2. The analysis revealed that the following factors were independent predictors of MACE occurrence:

• Killip class IV (OR = 3.758, *P* *=* 0.009)
• High levels of sST2 (OR = 1.008, *P* *=* 0.009)
• High levels of LVEDD (OR = 1.106, *P* *=* 0.009)
• High levels of LDL-C (mmol/L) (OR = 1.533, *P* *=* 0.041)
• Low levels of HALP score (OR = 0.958, *P* *=* 0.023). These factors were identified as significant contributors to the risk of MACE. The detailed results of the statistical analysis are presented in Table 2.

**Table 2:** Logistic regression analysis of factors influencing MACE occurrence.

Variables	B	SE	Wald x²	Odds Ratio (OR)	95%CI	*P*
Killip classification						
Class I				1.000		
Class II	0.265	0.358	0.545	1.303	0.645–2.631	0.460
Class III	0.956	0.827	1.336	2.603	0.514–13.172	0.248
Class IV	1.324	0.658	4.049	3.758	1.035–13.646	0.044
st2	0.008	0.003	6.761	1.008	1.002–1.014	0.009
Neutrophils	0.111	0.072	2.363	1.118	0.970–1.288	0.124
Serum creatinine	−0.005	0.004	1.381	0.995	0.987–1.003	0.240
LDL-C (mmol/L)	0.427	0.209	4.178	1.533	1.018–2.308	0.041
LVEDD	0.101	0.039	6.847	1.106	1.026–1.193	0.009
HALP Score	−0.043	0.019	5.135	0.958	0.924–0.994	0.023
SIRI	0.053	0.064	0.672	1.054	0.930–1.195	0.412
NLR	−0.130	0.084	2.376	0.878	0.744–1.036	0.123
PNI	−0.075	0.049	2.309	0.928	0.842–1.022	0.129
PLR	0.001	0.004	0.069	1.001	0.994–1.008	0.792

### 3.3 ROC curve analysis of independent predictors and combined indicators

Based on the results of the multivariate logistic regression analysis (with a significance level of *P* < 0.05), we identified five independent risk factors that are meaningful for predicting the risk of MACE within 1 year after PCI in patients with AMI: Killip classification, sST2, LDL-C, LVEDD, and HALP score. We performed ROC curve analysis for these five independent predictors and for the combined indicators.

The results showed that the AUC values for the individual predictors and the combined indicators were as follows:

• Killip classification: AUC = 0.654 (95% CI: 0.582–0.725)
• sST2: AUC = 0.732 (95% CI: 0.666–0.797)
• LDL-C: AUC = 0.579 (95% CI: 0.506–0.652)
• LVEDD: AUC = 0.607 (95% CI: 0.533–0.681)
• HALP score: AUC = 0.731 (95% CI: 0.667–0.795)
• Combined indicators: AUC = 0.833 (95% CI: 0.781–0.886)

The predictive performance, ranked from highest to lowest, was as follows: combined indicators > sST2 > HALP score > Killip classification > LVEDD > LDL-C.

**Figure 1:**
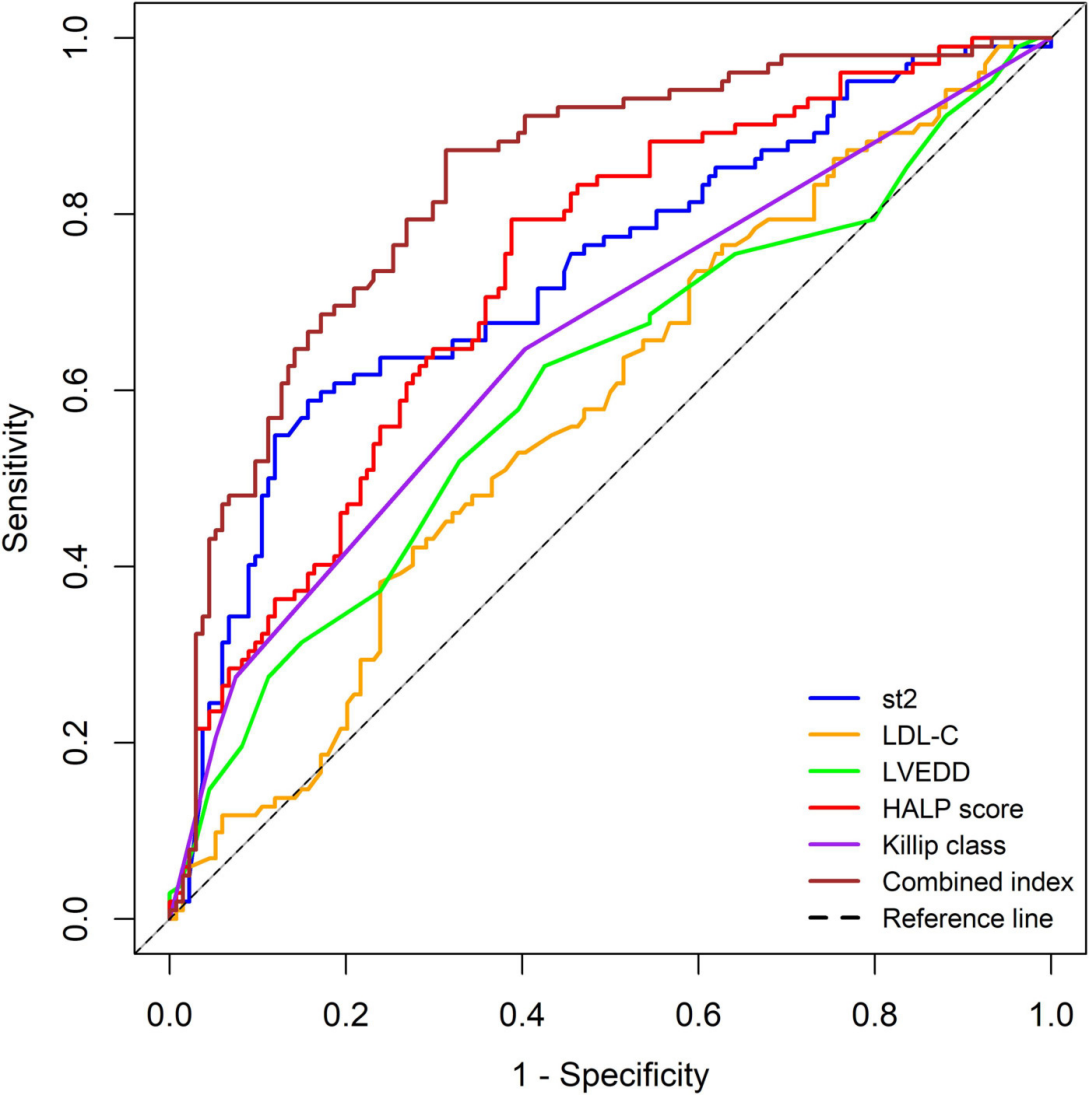
ROC curves for independent predictors and combined indicators.

The detailed results are shown in Table 3, and the ROC curves are presented in Figure 1.

**Table 3:** Assessment of predictive performance for MACE occurrence by independent predictors and combined indicators.

Variable	AUC (95% CI)	Optimal cutoff value	Youden index	Sensitivity	Specificity	*P*
Killip classifications	0.654 (0.582–0.725)	1.500	0.244	0.647	0.597	<0.001
st2	0.732 (0.666–0.797)	72.470	0.431	0.588	0.843	<0.001
LDL-C (mmol/L)	0.579 (0.506–0.652)	3.655	0.146	0.422	0.724	0.038
LVEDD	0.607 (0.533–0.681)	47.350	0.202	0.627	0.575	0.005
HALP score	0.731 (0.667–0.795)	35.666	0.406	0.794	0.612	<0.001
Combined indicators	0.833 (0.781–0.886)	0.339	0.560	0.873	0.687	<0.001

### 3.4 Nomogram prediction model for MACE occurrence

**Figure 2:**
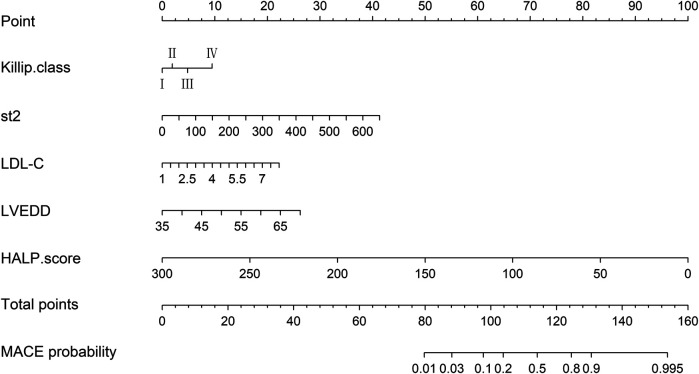
Nomogram for predicting the probability of MACE occurrence.

Based on the independent predictors of MACE occurrence—Killip classification, sST2, LDL-C, LVEDD, and HALP score—we constructed a nomogram prediction model for MACE occurrence (Figure 2). This nomogram allows for the relatively rapid estimation of MACE risk for each patient by assigning specific scores (Points) to each of the five independent predictive factors: Killip classification, sST2, LDL-C, LVEDD, and HALP score. The total score (Total Points) is obtained by summing the scores of these five factors, and each total score corresponds to a specific probability of MACE occurrence (Predicted Value).

The detailed nomogram model is presented in Figure 2.

### 3.5 Validation of the nomogram prediction model for MACE occurrence

The nomogram prediction model for MACE occurrence was validated using the Bootstrap resampling method (*N* = 1000). The model's predictive performance and consistency were comprehensively assessed using ROC curves and calibration curves.

**Figure 3:**
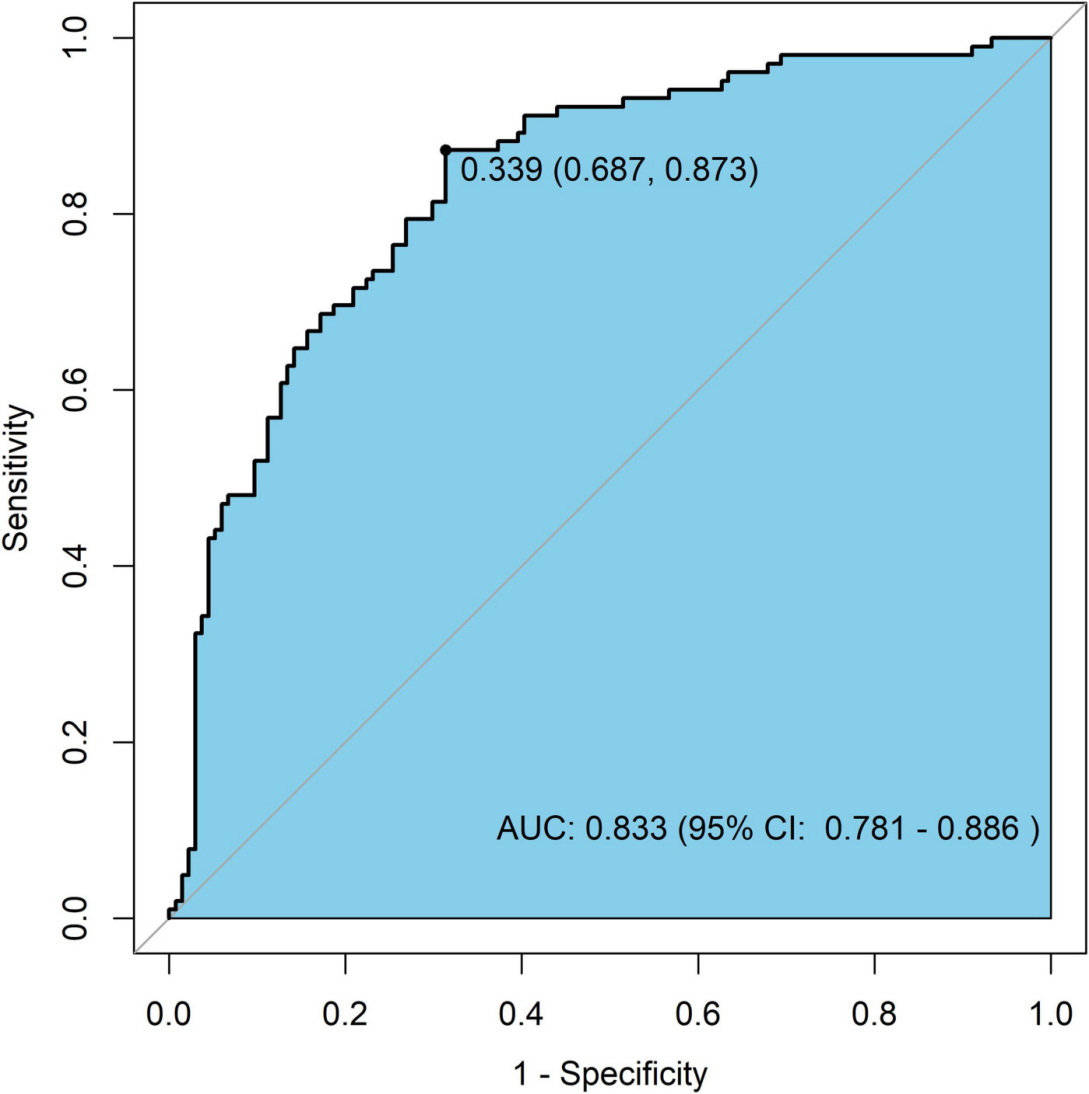
ROC curve of the nomogram prediction model for MACE occurrence.

Discrimination Ability: The ROC curve for the nomogram prediction model of MACE occurrence is shown in Figure 3. The model achieved an AUC of 0.833 (95% CI: 0.781–0.886), indicating high accuracy and discrimination ability.

**Figure 4:**
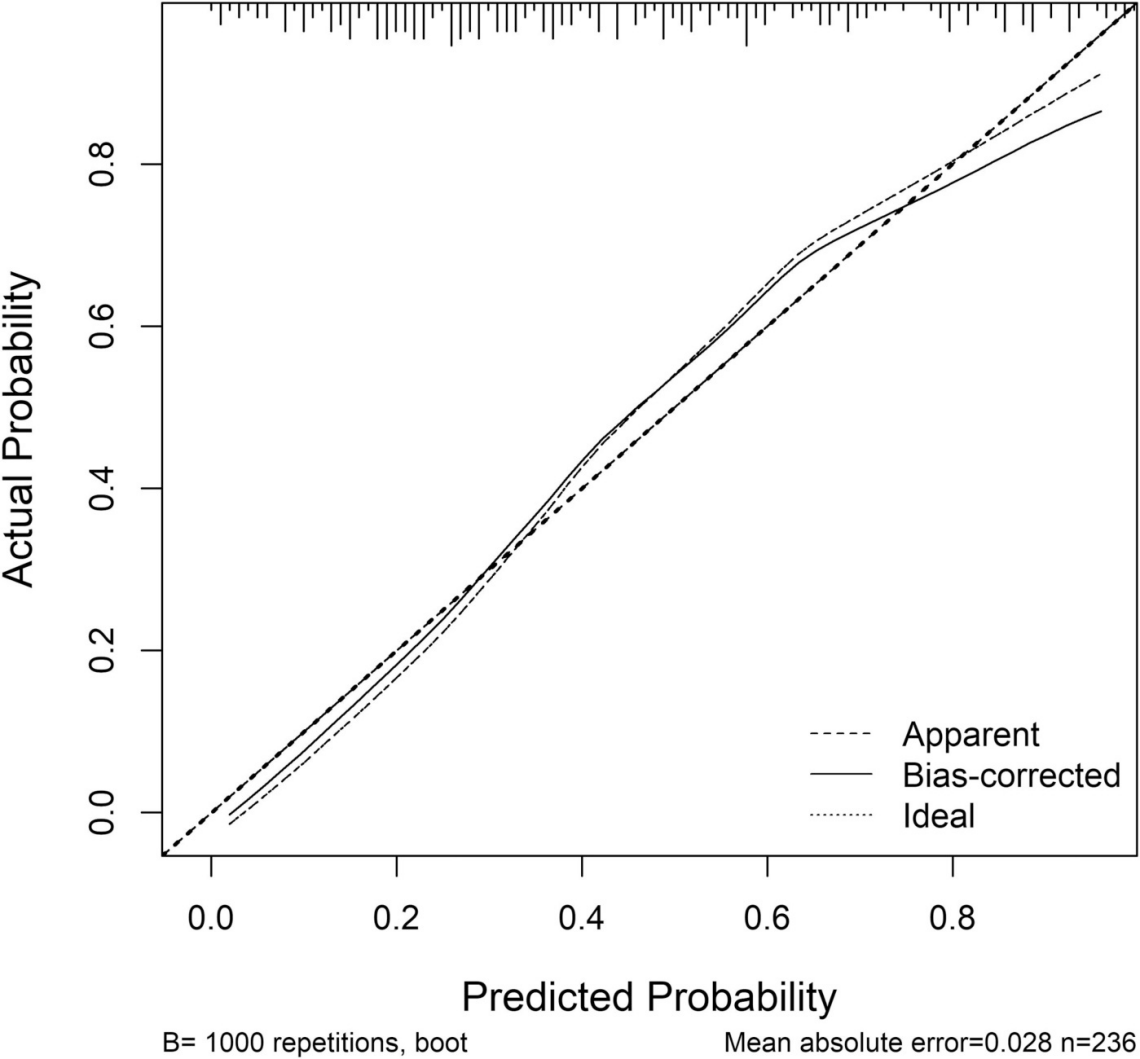
Calibration curve of the nomogram prediction model for MACE occurrence.

Calibration: The calibration curve for the nomogram prediction model is shown in Figure 4. The Hosmer-Lemeshow (HL) test result showed that the model's predicted probability was not significantly different from the actual probability of occurrence ($x^{2}$ *=* 11.864, *P* *=* 0.157, *P* > 0.05), suggesting good calibration.

## 4 Discussion

This study retrospectively analyzed data from 236 patients with AMI to develop and validate a nomogram prediction model that integrates the HALP score, sST2, Killip classification, cardiac structural parameter LVEDD, and lipid parameter LDL-C. The main findings include: (1) Independent Predictive Value of Novel Biomarkers: Low HALP score (OR = 0.958, *P* *=* 0.023) and high sST2 levels (OR = 1.008, *P* *=* 0.009) were identified as independent predictors of MACE within 1 year after PCI. Their predictive performance (AUC: HALP *=* 0.731, sST2 = 0.732) demonstrated significant efficacy, surpassing traditional indicators such as LDL-C (AUC = 0.579) and LVEDD (AUC = 0.607). Killip class IV (OR = 3.758) and increased LVEDD (OR = 1.106) further confirmed the importance of hemodynamic disturbances. (2) Synergistic Effect of Multiple Indicators: The combined model (HALP + sST2 + Killip + LVEDD + LDL-C) exhibited excellent predictive performance (AUC = 0.833, 95% CI: 0.781–0.886), with a sensitivity of 87.3% and specificity of 68.7%. This confirmed the synergistic predictive value of integrating inflammation, fibrosis, and clinical characteristics. (3) Model Validation Results: The nomogram demonstrated good calibration (Hosmer-Lemeshow test *P* *=* 0.157) and clinical applicability through internal validation using Bootstrap resampling (*N* = 1,000). This study provides the first visual risk assessment tool for East Asian AMI patients that integrates nutritional, inflammatory, and myocardial stress biomarkers.

AMI is a severe form of coronary artery disease, most commonly caused by the rupture, erosion, or ulceration of primary coronary plaques. These events lead to thrombus formation and subsequent reduction or interruption of coronary blood flow, resulting in acute ischemic necrosis of the myocardium (21, 22). For patients with AMI, especially those with ST-segment elevation myocardial infarction (STEMI), PCI remains the most common and effective treatment. However, patients still face the risk of MACE after PCI, which can be life-threatening. Studies have shown that changes in serum biomarkers reflect acute events such as endothelial injury, platelet activation, aggregation, and thrombus formation, all of which are components of the progression of coronary atherosclerosis. These biomarkers can effectively predict the occurrence of MACE after surgery (23, 24). Platelets, derived from megakaryocytes in the bone marrow, play an important role in hemostasis and the development of atherosclerotic complications. Research by Tsai et al. has found that both increases and decreases in platelet counts are associated with adverse cardiovascular outcomes (25, 26). Albumin, the most abundant protein in the blood, accounting for 50% of plasma proteins, not only reflects nutritional status but has also been shown to be associated with poor in-hospital outcomes in STEMI patients when serum albumin levels are low (27, 28). Following myocardial infarction, a large number of inflammatory mediators are released into the bloodstream, triggering an inflammatory response (29). Lymphocytes, early markers of physiological stress, can inhibit excessive immune responses and limit myocardial damage. Wang et al. found that lymphocytes play a key regulatory role in the inflammatory response after acute coronary syndrome (ACS) (30, 31). Hartopo et al. demonstrated that anemia may lead to poor prognosis in patients with ACS, with hemoglobin being a hematological biomarker reflecting anemia (32). In our study, the HALP score integrates the above four indicators (hemoglobin, albumin, lymphocytes, and platelets) to investigate the risk of MACE within one year after PCI in patients with acute myocardial infarction. In the comparative analysis between the two groups, the HALP score in the MACE group (median = 26.62) was significantly lower than that in the non-MACE group (median = 39.76) (*P* < 0.001), indicating an association between HALP score and MACE risk. Subsequent multivariate logistic regression analysis revealed that a low HALP score (OR = 0.958, *P* *=* 0.023) is an independent risk factor for MACE occurrence. In the ROC curve analysis, the AUC corresponding to the HALP score was 0.731 (95% CI: 0.667–0.795), indicating moderate independent predictive efficacy for MACE risk, consistent with existing research findings (33, 34).

The ST2 receptor is a member of the Toll-like/interleukin-1 receptor family. Upon activation of the IL-33/ST2 signaling pathway, the clinically detectable soluble ST2 (sST2) is released into the circulation. Elevated levels of sST2 have been associated with cardiac fibrosis, and studies by Kohli et al. have reported a strong correlation between ST2 and the risk of heart failure following myocardial infarction (35, 36). Current research on the prognostic value of ST2 in cardiovascular diseases has primarily focused on heart failure, with evidence suggesting that sST2 is an independent predictor of adverse events and mortality in heart failure patients (37). In contrast, studies on its predictive value for myocardial infarction prognosis are relatively limited. Weinberg et al. found that elevated sST2 levels in heart failure patients following acute myocardial infarction are associated with poor prognosis (38). In our study, we observed that sST2 levels in the MACE group (median = 87.51) were significantly higher than those in the non-MACE group (median = 37.53). The observed sST2 levels in our cohort (median 87.51 pg/ml in MACE group) were higher than previously reported ranges in AMI populations, which may reflect differences in assay methodology. We speculate that this may be related to its role as a decoy receptor, binding to IL-33 (a cardioprotective cytokine) and blocking the IL-33/ST2l pathway, which may exacerbate myocardial injury and fibrosis (39). Additionally, in our multivariate logistic regression analysis, elevated sST2 (OR = 1.008, *P* *=* 0.009) was identified as an independent risk factor for MACE occurrence. Similarly, in the ROC curve analysis, the AUC corresponding to sST2 was 0.732 (95% CI: 0.666–0.797), indicating moderate predictive efficacy for MACE risk.

In addition, during the baseline analysis of the two groups, we found that, apart from the HALP score and sST2, several other indicators such as Killip classification, neutrophils, serum creatinine, LDL-C, SIRI, NLR, PLR, and PNI showed significant differences (*P* < 0.05). These indicators include: (1) SIRI (Systemic Immune-Inflammation Index): Neutrophil count × Monocyte count/Lymphocyte count; (2) NLR (Neutrophil-to-Lymphocyte Ratio): Neutrophil count/Lymphocyte count; (3) PLR: Platelet count/Lymphocyte count; (4) PNI: Serum albumin (g/L) + 5 × Lymphocyte count (×10⁹/L). These are all immune-inflammatory indicators. The significant differences suggest that the risk of MACE within 1 year after PCI in patients with acute myocardial infarction is related to inflammatory or nutritional status, which is consistent with previous studies (9, 40, 41). However, in the multivariate analysis, these inflammatory indicators were not significant, which we speculate may be related to the sample size and warrants further investigation in our subsequent studies. Some studies have shown that compared with patients with postoperative LDL-C < 1.4 mmol/L, the risk of MACCE is still higher in patients with LDL-C ≥ 1.8 mmol/L and those with LDL-C between 1.4 and < 1.8 mmol/L (42). Our study revealed that in the MACE group, pre-PCI LDL-C levels (median = 3.47 mmol/L) were significantly higher than those in the non-MACE group (median = 3.29 mmol/L). This indicates that the baseline lipid levels of patients are associated with MACE risk. In the multivariate analysis, high levels of LDL-C (OR = 1.533, *P* *=* 0.041) were identified as an independent risk factor. Ischemia following myocardial infarction leads to myocardial necrosis and fibrosis, and left ventricular dilation is a core manifestation of ventricular remodeling, affecting the long-term prognosis of patients with myocardial infarction. LVEDD, as an easily obtainable imaging parameter, holds significant value for risk stratification and individualized management after PCI. Our study also confirmed its correlation with MACE risk and its independent predictive ability.

We performed ROC curve analysis for the five independent predictors identified in the multivariate logistic regression analysis, as well as for the combined indicators. The results showed that the predictive performance, ranked from highest to lowest, was as follows: combined indicators > sST2 > HALP score > Killip classification > LVEDD > LDL-C. Notably, the HALP score (AUC = 0.731) and sST2 (AUC = 0.732) demonstrated similar predictive capabilities. However, they have distinct biological significances: the HALP score reflects a systemic imbalance of inflammation and nutrition, while sST2 indicates myocardial fibrosis stress response (13, 17). This biological heterogeneity suggests that the two may be involved in the pathogenesis of MACE through different pathways. The significant improvement in the combined model (AUC = 0.833) confirmed the necessity of integrating indicators from different pathological and physiological dimensions, especially in identifying high-risk patients (with a sensitivity of 87.3%). This is of great value in screening subgroups that require aggressive intervention.

Based on the ROC curve analysis, we further concluded that Killip classification, sST2, LDL-C, LVEDD, and HALP score are independent predictors of MACE risk within 1 year after PCI in patients with AMI. Moreover, the combined indicators demonstrated excellent predictive performance. Nomograms can visualize complex mathematical models, making prediction results more readable and assisting clinicians in better assessing patient prognosis (43). Current risk prediction models for AMI mainly include the TIMI score (based on clinical indicators such as age and Killip classification), the GRACE score (integrating electrocardiographic and biochemical indicators), and the CADILLAC score (specifically for post-PCI risk assessment). Although these models have clinical utility, they have limitations: the TIMI score inadequately covers inflammatory and metabolic indicators, the GRACE score relies on complex laboratory tests and is limited in its application in primary hospitals, and the CADILLAC score does not incorporate emerging biomarkers (such as sST2 and HALP score) (44–48). Compared to traditional TIMI/GRACE scores, this model integrates various indicators such as myocardial fibrosis (measured by sST2), inflammatory and nutritional status (HALP), and cardiac remodeling (LVEDD). For patients with significantly reduced HALP scores, which indicate malnutrition combined with chronic inflammation, nutritional support can start alongside anti-inflammatory treatment. Those with high sST2 levels, which means active myocardial fibrosis, might need more aggressive treatment to reverse remodeling. The nomogram model developed in this study shows significant value in clinical application by integrating HALP score, sST2, and standard clinical indicators [such as Killip classification, left ventricular end-diastolic diameter (LVEDD), and low-density lipoprotein cholesterol (LDL-C)]. This model helps us accurately assess risk levels, with its combined prediction model (AUC = 0.833, 95% CI: 0.781–0.886) performing significantly better than individual indicators (HALP score AUC = 0.731; sST2 AUC = 0.732), letting us easily figure out each patient's risk of major cardiovascular events (MACE). For example, patients at high risk who have sST2 levels over 87.51 pg/ml and HALP scores under 26.62 have a significantly higher risk of MACE compared to low-risk populations, which is shown in the comparison of median values. Furthermore, the model can help shape intervention strategies; for patients with lower HALP scores, they might need better anti-inflammatory and nutritional support, while elevated sST2 levels indicate ongoing heart muscle scarring, suggesting that these patients could really benefit from early anti-reconstruction therapy, such as the use of angiotensin receptor-neprilysin inhibitors (ARNI) or sodium-glucose cotransporter 2 inhibitors (SGLT-2 inhibitors). For patients with Killip classification reaching level IV and increased LVEDD, we need to keep a close eye on their blood flow changes. Most importantly, all indicators of this model are part of the routine preoperative assessments for PCI, without any extra costs, making it a great fit for use in primary care hospitals. The nomogram developed in this study is the first AMI risk tool to integrate inflammatory-metabolic (HALP) and myocardial stress (sST2) indicators with clinical parameters, and its visual design aids in individualized treatment decision-making.

This study has limitations. First, the single-center retrospective design can introduce selection bias. This is a key limitation of single-center studies; retrospective designs cannot fully control for confounding variables. Although known covariates were adjusted for using multivariable analysis, unmeasured potential confounders (such as medication adherence and socioeconomic factors) could still influence the results. The data for this study came from the case system from a single medical institution, which is a regional tertiary hospital, where the patient population might have regional specificity in genetic background, dietary habits, and access to medical resources. Secondly, the study did not assess the dynamic changes in HALP scores and their impact on prognosis. Furthermore, the study utilized a relatively small sample size for internal validation, and the model's generalizability needs further validation through multicenter prospective studies and larger external cohorts. Finally, this single-center model needs external validation in multicenter cohorts. Future research should compare its performance against established risk scores, like GRACE 2.0 or TIMI risk scores, in a prospective setting.

## 5 Conclusion

The nomogram model developed in this study, which integrates the HALP score, sST2, and traditional clinical indicators (Killip classification, LVEDD, LDL-C), effectively predicts the risk of MACE within 1 year after PCI in patients with acute myocardial infarction. This tool offers the following clinical benefits: (1) Relatively Precision Stratification: Identifies high-risk patients who require intensified interventions (e.g., sST2 > 72.47 ng/ml or HALP < 35.66). (2) Comprehensive Pathophysiological Coverage: Simultaneously assesses inflammatory-metabolic imbalances and cardiac remodeling. (3) Operational Convenience: Based on routine testing indicators, suitable for rapid decision-making in outpatient and emergency settings. Future work should involve validation through multicenter cohorts and exploration of whether interventions targeting HALP/sST2 levels can improve prognosis.

## Data Availability

The original contributions presented in the study are included in the article/Supplementary Material, further inquiries can be directed to the corresponding author.
